# AP-64, Encoded by *C5orf46*, Exhibits Antimicrobial Activity against Gram-Negative Bacteria

**DOI:** 10.3390/biom11040485

**Published:** 2021-03-24

**Authors:** Kunhong Zhong, Yuelong Wang, Zeng Wang, Zongliang Zhang, Shasha Zhao, Hexian Li, Jianhan Huang, Wenhao Guo, Xi Zheng, Gang Guo, Liangxue Zhou, Hui Yang, Aiping Tong

**Affiliations:** 1 State Key Laboratory of Biotherapy and Cancer Center, West China Hospital, West China Medical School, Sichuan University, Chengdu 610041, China; zhongkunhong1993@126.com (K.Z.); wangzengff1996@163.com (Z.W.); zhangzongliang2019@163.com (Z.Z.); zhaoss_vz@163.com (S.Z.); harmony4067@163.com (H.L.); bruce212@126.com (W.G.); guogang@scu.edu.cn (G.G.); 2 Department of Neurosurgery, West China Hospital, Sichuan University, Chengdu 610041, China; yuelongwang@scu.edu.cn (Y.W.); jamy888@126.com (J.H.); liangxue_zhou@126.com (L.Z.); 3 Lung Cancer Center, West China Hospital, Sichuan University, Chengdu 610041, China; rmhahea@ucl.ac.uk; 4 Department of Otolaryngology, Head and Neck Surgery, West China Hospital, Sichuan University, Chengdu 610041, China

**Keywords:** antimicrobial peptides, AMPs, Gram-negative bacteria, *C5orf46*, AP-64

## Abstract

Antimicrobial peptides (AMPs), which are evolutionarily conserved components of the innate immune response, contribute to the first line of defense against microbes in the skin and at mucosal surfaces. Here, we report the identification of a human peptide, encoded by the chromosome 5 open reading frame 46 (*C5orf46*) gene, as a type of AMP, which we termed antimicrobial peptide with 64 amino acid residues (AP-64). AP-64 is an anionic amphiphilic peptide lacking cysteines (MW = 7.2, PI = 4.54). AP-64 exhibited significant antibacterial activity against Gram-negative bacteria, including *Escherichia coli* DH5α, *Escherichia coli* O157:H7, *Vibrio cholerae*, and *Pseudomonas aeruginosa*. Moreover, AP-64 was efficient in combating *Escherichia coli* O157:H7 infections in a mouse model and exhibited cytotoxic effects against human T-cell lymphoma Jurkat and B-cell lymphoma Raji cells. We also observed that Gm94, encoded by mouse *C5orf46* homologous gene, closely resembles AP-64 in its antibacterial properties. Compared with other human AMPs, AP-64 has distinct characteristics, including a longer sequence length, absence of cysteine residues, a highly anionic character, and cell toxicity. Together, this study identified that AP-64 is an AMP worthy of further investigation.

## 1. Introduction

Bacteria live in enormous numbers in almost every environment on earth, from deep-sea vents to the digestive tracts of humans. As important decomposers in the earth’s ecosystem, bacteria are responsible for breaking down dead plants and animals into organic compounds. However, bacterial pathogens pose one of the most urgent global health threats because of their growing resistance to current antibacterial drugs [1]. Compared with Gram-positive bacteria, the Gram-negative bacteria are harder to kill because of the presence of the outer membrane, which contributes to the prevention of the penetration of antibacterial drugs into the cells [2]. Infections caused by drug-resistant bacteria have aroused wide concern because of the lack of effective antimicrobial agents [3]. Compared with conventional antibiotics, antimicrobial proteins (AMPs) directed to the bacterial membrane are assumed to be more effective at targeting bacteria and preventing resistance [4].

AMPs are evolutionarily conserved components of the innate immune response that provide host defense at the skin and mucosal surface [5]. The human AMPs identified to date include mainly histatins, hepcidins, defensins, and LL-37. Histatins, which are found in human saliva, possess antifungal properties by binding to a receptor on the fungal cell membrane [6]. Hepcidins are produced by the human liver and serve as potential antibacterial and antifungal agents [7]. Human defensins, including α-defensins and β-defensins, contribute to the antimicrobial action of granulocytes, the mucosal host defense in the small intestine, as well as the epithelial host defense in the skin and elsewhere [8]. LL-37 is a 37-residue, amphipathic, helical peptide that is found throughout the body and exhibits a broad spectrum of antimicrobial activities [9]. Moreover, increasing evidence has demonstrated that AMPs have many other biological and physiological functions. For example, LL-37 not only exerts a bactericidal effect but also plays an important role in immune modulation and wound healing [10].

Human genome projects have produced a large amount of genomic sequence data. However, the biological functions of many proteins encoded by the sequences obtained remain unknown. Functional research on these proteins may help us uncover novel molecular pathways and potential drug targets. Previously, we identified an uncharacterized peptide, chromosome 10 open reading frame 99 (*C10orf99*), as a type of human AMP, which was termed AP-57 [11]. Here, using a bioinformatics analysis, we found that another gene, chromosome 5 open reading frame 46 (*C5orf46*), encodes an amphiphilic small secretory peptide. Through prokaryotic expression and purification, as well as an antibacterial assay, *C5orf46* was identified as a type of AMP.

## 2. Materials and Methods

### 2.1. Cell Culture

The cell lines used in this study were purchased from the American Type Culture Collection (ATCC). The cells were cultured in DMEM or RPMI 1640 medium containing 10% fetal bovine serum (Gibco, Grand Island, NY, USA) and 100 μg/mL penicillin–streptomycin (ZS808; Zomanbio, Beijing, China) at 37 °C in a humidified atmosphere of 5% CO_2_.

### 2.2. Expression and Purification of Recombinant Proteins

The sequences of human *C5orf46* (AP-64) and mouse *C5orf46* (Gm94 named by Harney et al.) [12] were obtained from the National Center for Biotechnology Information database and synthesized by GENEWIZ (Beijing, China). A small ubiquitin-related modifier (SUMO) tag was fused to the N terminus of AP-64 or Gm94. The fragments were subcloned into the pET28a vector (69864-3; Addgene, Watertown, MA, USA) to construct the recombinant plasmids pET28a-SUMO-AP-64 and pET28a-SUMO-Gm94. Recombinant protein expression and purification were performed generally according to a previously reported method [13]. Briefly, the recombinant plasmid was transformed into *E. coli* Rosetta (DE3) cells. The cells were cultured in lysogeny broth (LB) medium at 37 °C to an optical density at 600 nm (OD600) of 0.6–0.8. Isopropyl β-d-1-thiogalactopyranoside (IPTG, 1 mM) was added to the culture to induce recombinant protein expression. The cells were harvested and lysed by sonication 16 h after induction. The lysates were fractionated by centrifugation at 15,000 rpm for 15 min at 4 °C. The supernatant was applied to a Ni-nitrilotriacetate (Ni-NTA) Sepharose column, and 6× His-tagged SUMO-AP-64 or SUMO-Gm94 was eluted using elution buffer (500 mM NaCl and 250 mM imidazole, pH 8.0). The fusion proteins diluted in phosphate-buffered saline (PBS) at a final concentration of 1 mg/mL were cleaved by SUMO protease (12588018; Invitrogen, Shanghai, China) at 4 °C for 6 h. The cleaved samples were reloaded onto the Ni-NTA resin to obtain the AP-64 or Gm94 protein. The protein concentration was measured with a BCA (P0012S; Beyotime, Shanghai, China) assay kit according to the manufacturer’s instructions.

### 2.3. Antibacterial Activity Detection of Purification Proteins

*E. coli* DH5α cells were purchased from Invitrogen (18288019; Shanghai, China) for antibacterial activity detection of purified proteins. After overnight growth in LB at 37 °C, *E. coli* DH5α cells were washed and diluted in LB to an OD600 of 0.1. After adding SUMO-AP-64 (21 μg/mL), SUMO-Gm94 (21.4 μg/mL), AP-64 (7.2 μg/mL), or Gm94 (7.6 μg/mL) (1 μM, the same order of magnitude as known for other AMPs) to DH5α cells (100 µL), bacteria were incubated at 37 °C for 4 h and then diluted with LB. The 100 µL samples of the dilutions were plated onto LB agar plates and incubated at 37 °C for 16 h. The final number of colony-forming units (CFUs) was counted.

Meanwhile, a zone of inhibition test of the proteins against *E. coli* DH5α was performed using the Oxford cup method [14]. Briefly, 100 μL of diluted inoculum (10^5^ CFU/mL) from bacteria suspensions was added to and spread on the surface of LB agar plates. Sterilized Oxford cups (Φ 5 mm) were then placed on the agar medium and filled with 100 µL of SUMO-AP-64, SUMO-Gm94, AP-64, or Gm94 protein (1 μM); the plates were incubated at 37 °C for 18 h. An equivalent volume of PBS was used as a control.

### 2.4. Antibacterial Activity of AP-64 and Gm94 against DH5α

To evaluate the inhibition effect of different concentrations of AP-64 and Gm94 on growth of DH5α, the bacteria were cultured in LB at 37 °C until the OD600 reached 0.5 and were then diluted 10-fold. Subsequently, 100 μL of the cell culture was added to each well of microtiter plates containing LB medium supplemented with AP-64 or Gm94 (0.1–10 μM). After incubation at 37 °C for 8 h, OD600 measurements were performed on a universal microplate spectrophotometer (BioTek, Winooski, VT, USA).

Next, growth curve analysis was implemented. DH5α was exposed to AP-64, Gm94, and LL-37 (61302; AnaSpec, Beijing, China) at a concentration of 10 μM for 8 h, and the OD600 values were measured every 2 h. DH5α was used as the control.

### 2.5. SEM Detection for DH5α Cells

DH5α cells were treated with the proteins at a concentration of 10 μM and subjected to scanning electron microscopy (SEM) according to a method reported previously [15]. Briefly, treated bacteria were harvested at different time points and centrifuged at 5000× *g* for 4 min. The bacteria were fixed with 2% glutaraldehyde for 2 h before a thorough wash with PBS buffer. The fixed bacteria were dehydrated through an ascending alcohol series (30%, 50%, 65%, 85%, 90%, and 100% EtOH) for 10 min each. After a critical-point drying, the samples were visualized using SEM. DH5α cells treated with PBS were used as the blank control.

### 2.6. Antibacterial Activity against Pathogenic Bacteria

*Escherichia coli* O157:H7 (ATCC, 43895), *Vibrio cholerae* (ATCC, 51394), *Pseudomonas aeruginosa* (ATCC, 27853), *Staphylococcus aureus* (ATCC, 25923), and *Listeria monocytogenes* (ATCC, 19115) cells were treated using the method described above.

The minimum inhibitory concentrations of AP-64 or Gm94 were measured in the concentration range of 0.2–200 μM by standard double dilution methods [16] and were taken as the lowest concentration of peptide at which no visible growth was observed (Table S1).

### 2.7. Antibacterial Activity against Escherichia coli O157:H7 in Mouse

Male BALB/c mice (6 weeks, 18–22 g) were obtained from GemPharmatech (Jiangsu, China). Mice were randomly assigned to 4 groups with 10 mice in each group. O157:H7 (5 × 10^6^ CFU/mouse) was administered intraperitoneally to the mice. Thirty minutes later, the mice were injected intraperitoneally with AP-64, Gm94, or LL-37 at the dose of 500 μg/kg. An equivalent volume of PBS was used as a control. All animal experiments were approved by the West China Hospital of Sichuan University Biomedical Ethics Committee (ethic approval document: 2020166A), and all experiments conformed to all relevant regulator standards.

### 2.8. Antitumor Capacity

Human tumor cells (2 × 10^4^) were cultured in RPMI 1640 or DMEM medium containing 5% fetal bovine serum with AP-64 or Gm94 at the indicated concentration. After treatment at 37 °C for 24 h, cells were stained using a Cell Counting Kit-8 (CCK-8) according to the manufacturer’s protocol (C0037; Beyotime, Shanghai, China). The following formula was used to calculate the inhibition rate of cell growth (%): cell growth inhibition (%) = (1 − (percentage of surviving cells in the AP-64 or Gm94 treatment group/percentage of surviving cells in the PBS control group)) × 100. PBS-treated cells served as a control in all experiments.

### 2.9. Statistical Analysis

Data are expressed as the mean ± standard deviation from three independent experiments. Statistical analyses were performed using the GraphPad Prism Software version 5.0. For in vitro analysis, differences between experimental conditions were analyzed using unpaired Student’s *t*-test. Significance was set at *p* < 0.05. For in vivo data, a survival curve of mice was obtained using the Kaplan–Meier plot with a log-rank test. Significance was set at *p* < 0.05.

## 3. Results

### 3.1. Amino Acid Sequence Features of AP-64/C5orf46

**Figure 1:**
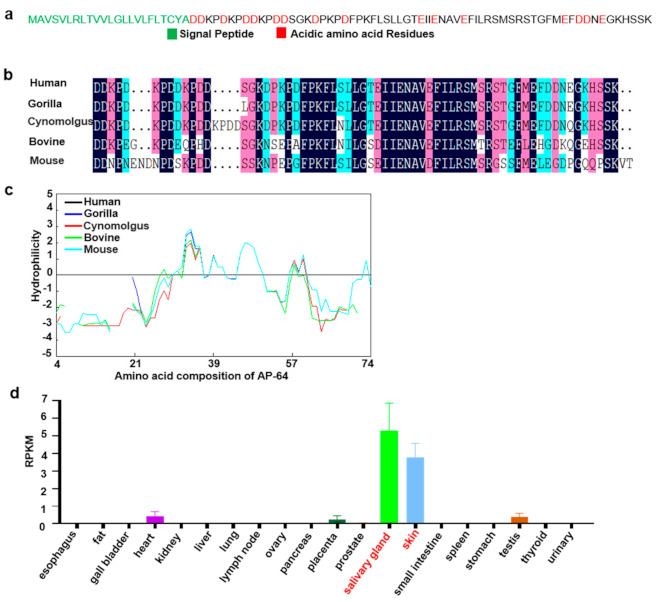
Features of the AP-64 amino acid sequence. (**a**) Amino acid sequence of AP-64. The signal peptide was predicted by UniProt. (**b**) Sequence conservation analysis. (**c**) Hydrophilicity analysis. (**d**) Expression analysis of the AP-64 mRNA in the TCGA database (https://www.ncbi.nlm.nih.gov/gene/389336, accessed on 12 October 2020).

The mature human *C5orf46* (AP-64) is a short acidic peptide (MW = 7.2, PI = 4.54, Figure 1a). A homology analysis showed that its acidic amino acid residues and hydrophobic region are conserved among several mammals, including common model animals such as the gorilla, cynomolgus, bovine, and the mouse (Figure 1b). A hydrophobicity analysis revealed that AP-64 is an amphiphilic peptide with a middle hydrophobic region and hydrophilic terminals (Figure 1c). The secondary structure prediction shows that an α helix is located in the hydrophobic region (http://bioinf.cs.ucl.ac.uk/psipred/, accessed on 15 February 2021) (Figure S1). The mRNA expression profile of AP-64 was generated using the TCGA database (https://www.ncbi.nlm.nih.gov/gene/389336, accessed on 12 October 2020). As shown in Figure 1d, AP-64 is mainly expressed in the salivary glands and skin.

### 3.2. Expression and Purification of Recombinant Proteins

**Figure 2:**
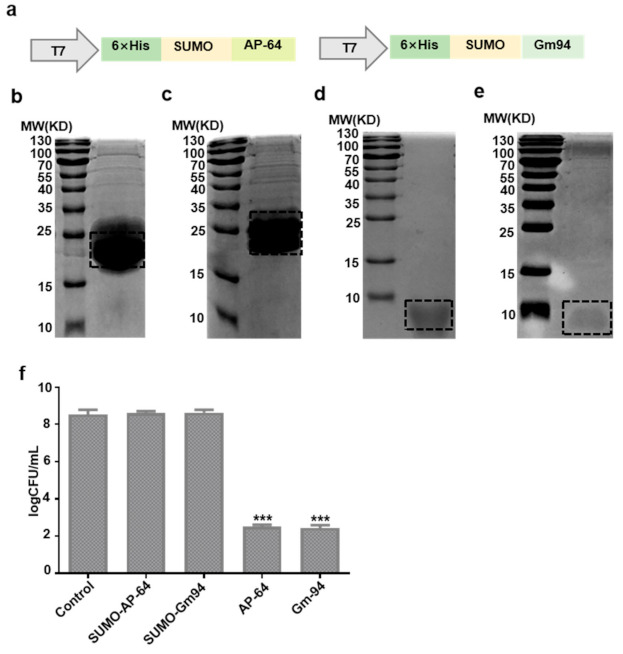
Expression and purification of purification proteins. (**a**) Schematic drawing of the constructs used for recombinant protein expression. (**b**–**e**) SDS–PAGE analysis of SUMO-AP-64, SUMO-Gm94, AP-64, and Gm94, corresponding to the expected molecular weight of 21 KD, 21.4 KD, 7.2 KD, and 7.6 KD, respectively. The protein positions are highlighted by the dotted line. (**f**) Effects of the proteins on the growth of *E. coli* DH5α. Bacteria were treated with the indicated agents at a concentration of 1 μM. Data are mean ± SD of three independent experiments with each experiment conducted in triplicate. *** *p* < 0.001, significantly different from the control.

SUMO-AP-64 and SUMO-Gm94 were expressed by a transformation of recombinant vectors into *E. coli* Rosetta (DE3)-competent cells after induction with IPTG (Figure 2a). The purified proteins were analyzed using sodium dodecyl sulfate–polyacrylamide gel electrophoresis (SDS–PAGE; Figure 2b,c). SUMO protease was used to remove the SUMO tag and obtain the target proteins. AP-64 and Gm94 were also analyzed using SDS–PAGE (Figure 2d,e). Circular dichroism (CD) spectrum analysis shows that AP-64 and Gm94 both possess an α-helix region as predicted (http://bioinf.cs.ucl.ac.uk/psipred, accessed on 15 February 2021) (Figure S2). In addition, the purified peptides were further verified by using chromatography and mass spectrometry (Figures S3 and S4).

Subsequently, DH5α cells were treated by the proteins, but only AP-64 or Gm94 treatment significantly inhibited the cell growth (Figure 2f). A zone of inhibition test against DH5α also showed the same result (Figure S5).

### 3.3. Antibacterial Activity against DH5α Cells

**Figure 3:**
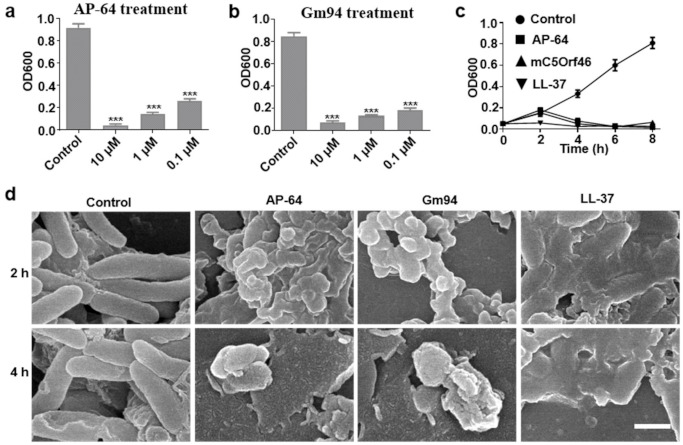
Antibacterial activity of AP-64 and Gm94 against DH5α cells. (**a**,**b**) OD600 value of DH5α cells exposed to the indicated concentrations of AP-64 and Gm94 for 8 h. Data are mean ± SD of three independent experiments with each experiment conducted in triplicate. *** *p* < 0.001. (**c**) OD600 value of DH5α cells treated with the indicated proteins (10 μM) at different time points. The proteins were added to the bacteria at time 0. All error bars represent standard deviation (SD). (**d**) SEM assay of DH5α cells after treatment with indicated proteins. DH5α cells were grown in LB to an OD600 of 0.1. The cells were treated with the indicated proteins (10 μM) at 37 °C. Scale bar, 1 μm.

We further evaluated the effects of different concentrations of AP-64 or Gm94 on DH5α cell growth. As shown in Figure 3a,b, after an 8 h treatment, AP-64 and Gm94 effectively inhibited the growth of bacteria in the concentration range of 0.1–10 μM. A growth curve analysis showed that DH5α cells exposed to AP-64 or Gm94 underwent a slow multiplication at the beginning of the culturing process (0–2 h), followed by a rapid decline. However, after 8 h of treatment, AP-64, Gm94, and LL-37 exhibited similar inhibition performances (Figure 3c).

SEM examination revealed that a 2-h treatment with the proteins at the concentration of 10 μM caused an apparent irregularity and collapse in cell shape. Extended treatment with AP-64 or Gm94 for 4 h caused serious cell damage (Figure 3d). After treatment for 6 h, the cells could barely be observed (Figure S6).

### 3.4. Antibacterial Activity against Pathogenic Bacteria

**Figure 4:**
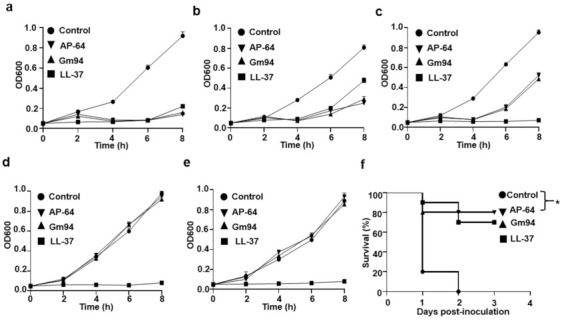
Antibacterial activity against pathogenic bacteria. (**a**–**e**) Growth curve of O157:H7 (**a**) *Vibrio cholerae*, (**b**) *Pseudomonas aeruginosa* (**c**), *Staphylococcus aureus* (**d**), and *Listeria monocytogenes* (**e**) cells exposed to the recombinant proteins (10 μM). The proteins were added to the bacteria at time 0. All error bars represent SD. (**f**) Enhanced resistance to O157:H7 infection after treatment with AP-64 or Gm94 in mice (n = 10 mice/group). Thirty minutes after the intraperitoneal inoculation of 5.0 × 10^6^ CFU of O157:H7, mice were injected intraperitoneally with the indicated proteins. The dosage of proteins was 500 μg/kg of body weight. A log-rank test was used to analyze the overall survival of the mice (* *p* < 0.05).

Following the identification that AP-64 and Gm94 significantly inhibited the DH5α cell growth, we detected antibacterial activity of the proteins against pathogenic bacteria. After a 4-h treatment with the proteins, only pathogenic Gram-negative bacteria (O157:H7, *Vibrio cholerae*, and *Pseudomonas aeruginosa* cells) growth was obviously inhibited (Figure S7). The growth curve analysis also indicated that Gram-negative bacteria exhibited susceptibility to AP-64 or Gm94 treatment. Nevertheless, AP-64 and Gm94 lacked appreciable bactericidal activity against the pathogenic Gram-positive bacteria *Staphylococcus aureus* and *Listeria monocytogenes* (Figure 4a–e). In addition, we also observed that the yeast cells were unsusceptible to the peptide treatment (Figure S8).

Next, we tested the ability of AP-64 and Gm94 to protect mice against an O157:H7 challenge at a lethal level. The survival rate of mice treated with PBS was 20% at 24 h after bacterial inoculation, whereas the survival rates of mice injected with AP-64 and Gm94 (500 μg/kg of body weight) were 90% and 80%, respectively (*p* < 0.05, Figure 4f).

### 3.5. Antitumor Capacity

**Figure 5:**
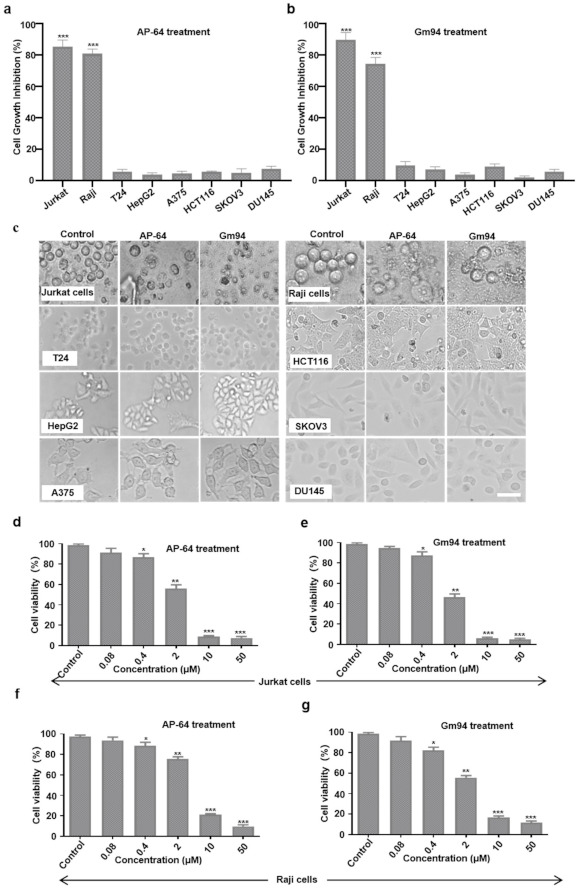
Antitumor activity. (**a**,**b**) Human tumor cells were treated with AP-64 (**a**) or Gm94 (**b**) at 10 μM for 24 h and cell growth inhibition was measured using the CCK-8 assay. (**c**) Representative images of the cells 24 h after treatment with AP-64 or Gm94 at 10 μM. The cell lines T24, HepG2, A375, HCT116, SKOV3, and DU145 were representative for Bladder cancer, liver cancer, melanoma, colon cancer, ovarian cancer, and prostate cancer tissues, respectively. (**d**–**g**) Cell viability of Jurkat or Raji cells after treatment with the proteins at indicated concentrations for 24 h. All error bars represent SD. * *p* < 0.05, ** *p* < 0.01, *** *p* < 0.001. Scale bar, 100 μm.

We also tested the potential antitumor effects of AP-64 and Gm94 in eight commonly used human cell lines. Jurkat (human T lymphoma cells) and Raji (human B lymphoma cells) cells exhibited susceptibility to AP-64 and Gm94 (10 μM) treatment, with almost complete disruption of the cells. The viability of Jurkat or Raji cells was significantly reduced after treatment with AP-64 or Gm94 at the concentration of 0.4 μM (Figure 5a–g). Nevertheless, these proteins lacked appreciable cytotoxic activity against the remaining tumor cell lines (Figure 5a–c). Subsequently, we tested the toxicity of the peptides to T cells, Hacat, and MEF cells. Our data showed that these cells were susceptible to the peptide treatment (Figures S9–S11).

## 4. Discussion

Despite the many efforts that have been devoted to the development of effective antibacterial agents, certain mammalian proteins with intrinsic antibacterial activity might be underappreciated. Here, we demonstrated that AP-64 (human *C5orf46*) is a type of AMP that exerts a direct antibacterial effect on Gram-negative bacteria, and our data shows that Gm94 (mouse *C5orf46)* also exhibits the similar antibacterial ability.

Although AMPs are promising candidates for the development of novel antibiotics, it is difficult to produce sufficient quantities of these proteins because of their toxicity toward microbial expression hosts. Previously, the SUMO tag was used widely for the prokaryotic expression of recombinant proteins, such as abaecin (a 34 amino acid long antimicrobial peptide from honeybees) [17]. In this study, the SUMO tag was fused to the N terminus of AP-64 to express the SUMO-AP-64 fusion protein. SUMO-AP-64 was expressed in a soluble form but failed to inhibit the growth of DH5α cells. After removal of the SUMO tag, AP-64 exhibited strong antibacterial effects. Thus, fusing a large tag, such as SUMO, to the N terminus of AP-64 blocked its antibacterial activity. We observed that bacteria treated with AP-64 underwent a slow multiplication at the beginning of the culturing process, followed by a rapid decline. This growth curve implied that AP-64 possessed a similar antibacterial pattern to that of cell-envelope-interfering agents and that its action might be related to cell envelope damage [18]. The multiplication of growth might also hint at an intracellular target of the peptide. It might need some time to become internalized into the cells before it can exert its antibacterial function.

A bioinformatics analysis using TCGA showed that AP-64 is present not only at the RNA level in the skin and salivary glands, but also in major blood vessels (aorta) and the heart [19]. Notably, mice that were heterozygous for a null mutation of Gm94 had a significantly increased fat mass and reduced lean mass compared with wild-type mice [20]. Recently, AP-64 was identified as a plasma protein through deep-proteome profiling of human plasma [12]. Thus, whether AP-64 plays a role in lipid homeostasis is worthy of further study.

AP-64 exhibits some distinct features compared with some human AMPs (defensins, LL-37, histatins, and hepcidin). First, compared with other human AMPs, which normally contain less than 45 amino acid residues, AP-64 consists of 64 residues. Second, while most of the human AMPs are highly cationic, AP-64 is an anionic peptide with a PI of 4.54. Third, unlike other human AMPs (such as defensins and hepcidin), AP-64 contains no cysteines. Meanwhile, AP-64 also exhibits similar features to other human AMPs. For example, both AP-64 and dermcidin have a net negative charge and contain no cysteines. In addition, similar to Microcin E492, AP-64 possesses activity against Gram-negative bacteria and tumor cells (https://wangapd3.com/main.php, accessed on 16 March 2021).

Although our study revealed that AP-64 exhibits strong antibacterial activity against Gram-negative bacteria, it had several limitations. First, the mRNA expression of AP-64 in the skin and salivary gland was discovered using the TCGA database; thus, a specific antibody should be generated, and this protein’s expression profile should be further investigated in detail by immunohistochemical staining. Second, the molecular mechanisms underlying the bacterial inhibition should be investigated in future studies. Third, our research only confirmed the antibacterial effect of AP-64 on the bacteria used in this study, but other bacteria and fungi should also be tested.

In summary, we demonstrated for the first time that AP-64/C5orf46 is a type of AMP. Compared with other human AMPs, AP-64 possesses distinctive characteristics, including a longer sequence length, absence of cysteine residues, highly anionic characteristics, and cell toxicity against Jurkat and Raji cells. Our data provided useful clues for further study of the physiological functions of AP-64/C5orf46.

## Data Availability

The data presented in this study are available in the article and Supplementary Material.

## Supplementary Materials

The following are available online at https://www.mdpi.com/2218-273X/11/4/485/s1: Table S1: Minimum inhibitory concentration values (µg/mL) of the antibacterial peptides. Figure S1: Prediction of the secondary structure with PSIPRED. Figure S2: Peptides corresponding to the predicted α helix when analyzed by CD spectroscopy. Figure S3: Size exclusion chromatography of the purified peptides. Figure S4: Mass spectra of the AP-64 and Gm94. Figure S5: Antibacterial ability test of indicated proteins toward DH5a cells using the Oxford cup method. Figure S6: Representative bright-field microscopic images of DH5a cells treated with different proteins (10 μM). Figure S7: Representative images of O157:H7, Vibrio cholerae, Pseudomonas aeruginosa, Staphylococcus aureus and Listeria monocytogenes cells after a 4-h treatment with indicated proteins at a concentration of 10 μM. Figure S8: Effect of AP-64 and Gm94 on yeast cell viability. Figure S9: Effect of AP-64 and Gm94 on T cell viability. Figure S10: Effect of AP-64 and Gm94 on Hacat cell viability. Figure S11: Effect of AP-64 and Gm94 on MEF cell viability.
