# Supplementary material for: AP-64, Encoded by C5orf46, Exhibits Antimicrobial Activity against Gram-Negative Bacteria
Source: Biomolecules. 2021 Mar 24;11(4):485. doi: 10.3390/biom11040485 (PMC8063792; doi:10.3390/biom11040485)
Supplement: Supplementary file 1 [file biomolecules-11-00485-s001.pdf]

Supplementary Material

Table S1. Minimum inhibitory concentration values (μg/mL) of the antibacterial peptides. −: no activity.

Microorganism	Name	AP-64	Gm94
Gram-negative bacterium	*Escherichia coli* O157:H7	3.6	3.26
	Vibrio cholerae	37.4	42.56
	Pseudomonas aeruginosa	66.24	65.1
Gram-positive bacterium	*Staphylococcus aureus*	-	-
	Listeria monocytogenes	-	-

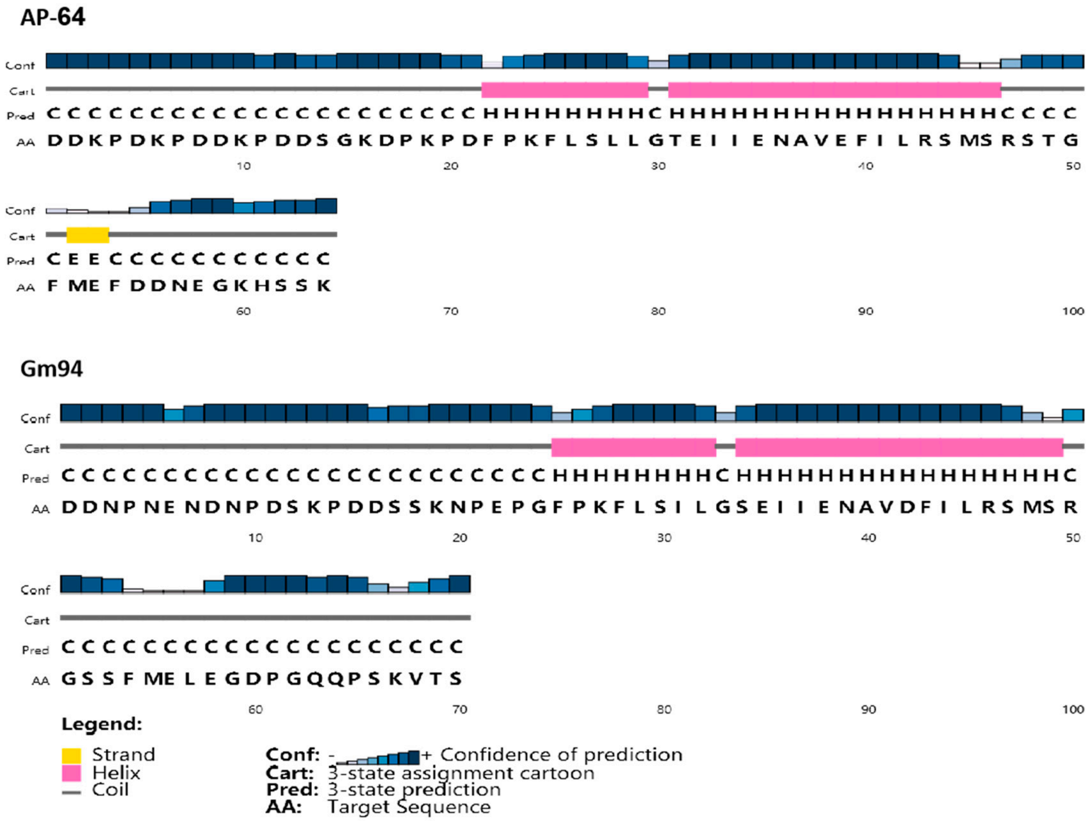

Figure S1. Prediction of the secondary structure with PSIPRED.

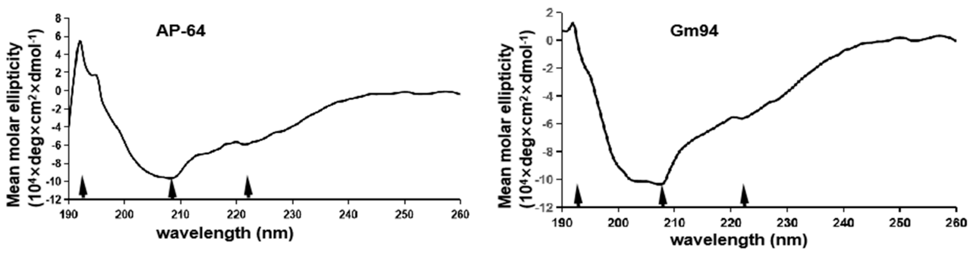

Figure S2. Peptides corresponding to the predicted α helix when analyzed by CD spectroscopy. CD spectra of the AP-64 and Gm94 peptide at a concentration of 200 μM in PBS (pH 7.0) at 25°C. The measured CD values were converted to mean residue molar ellipticities, [θ] mean, and plotted as a function of the incident wavelength. The arrowheads above the x axis identify the minima associated with an α-helical spectrum.

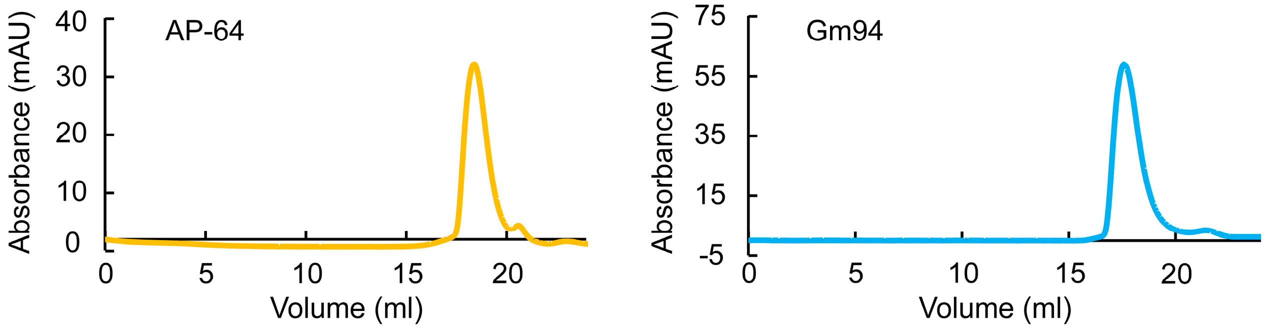

Figure S3. Size exclusion chromatography of the purified peptides. The 500 μL of peptide (3 mg/mL) was separated by a Superdex 200 column. HEPES buffered saline (20 mM HEPS, 100 mM NaCl) was used as the running buffer. The flow rate was 0.5 mL/min. Since AP-64 and Gm94 don't contain Tyr and Trp and the absorbance can not be measured at 280 nm, the absorbance was measured at 215 nm.

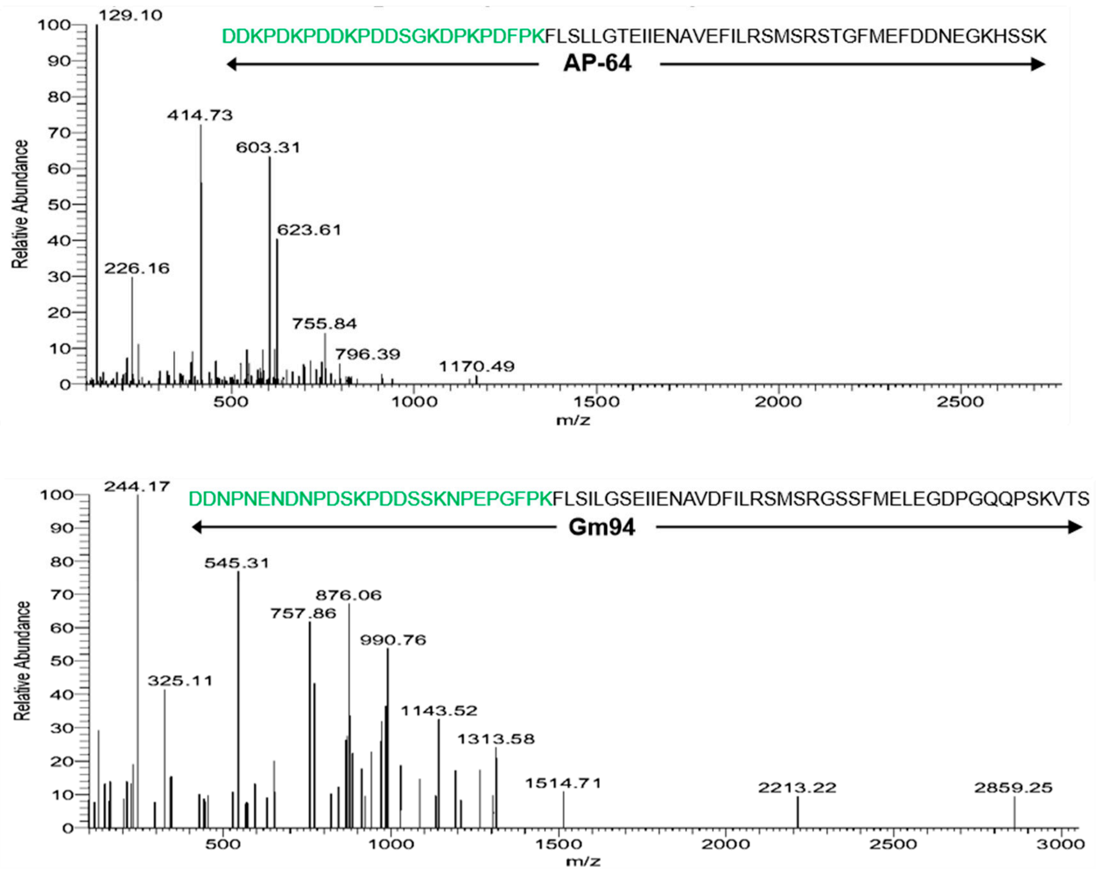

Figure S4 .Mass spectra analysis of AP-64 and Gm94. After SDS–PAGE analysis, the peptide bands were cut out from the Coomassie Blue-stained gels and digested with trypsin to generate peptide mixture. The peptide fragments were then analyzed by using Thermo Scientific Q Exactive Plus. The amino acid residues in green represent the positively identified region.

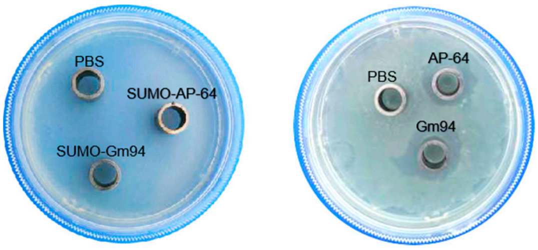

Figure S5. Antibacterial ability test of indicated proteins toward DH5a cells using the Oxford cup method. The concentration of the proteins used in the antibacterial ability test was 1 μM.

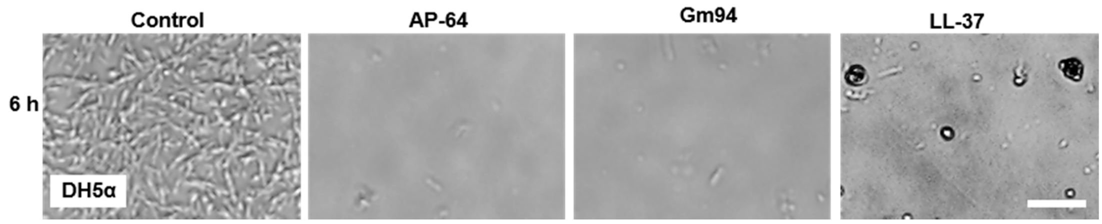

Figure S6. Representative bright-field microscopic images of DH5a cells treated with different proteins (10 μM). Scale bar, 100 μm.

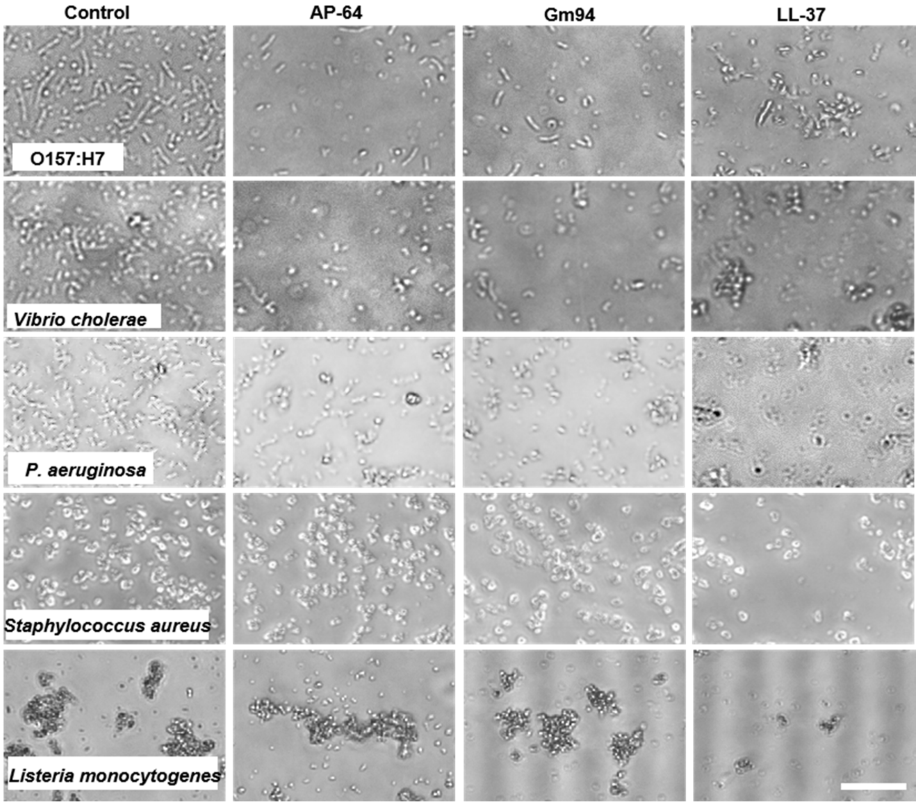

Figure S7. Representative images of O157:H7, Vibrio cholerae, Pseudomonas aeruginosa, Staphylococcus aureus and Listeria monocytogenes cells after a 4-h treatment with indicated proteins at a concentration of 10 μM. Scale bar, 100 μm.

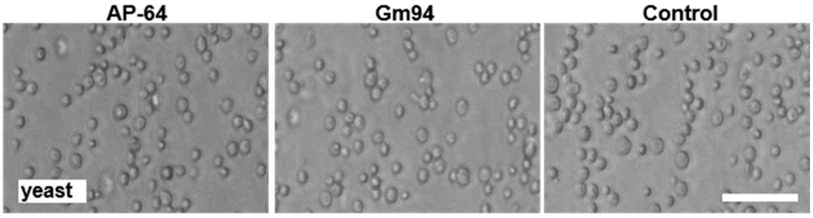

Figure S8. Effect of AP-64 and Gm94 on yeast cell viability. Representative images of yeast cells post treatment with indicated peptides. The yeast cells (6 × 10^4) were cultured with RPMI 1640 medium containing AP-64 or Gm94 (10 μM) at 37℃ for 24 h. Scale bar, 100 μm.

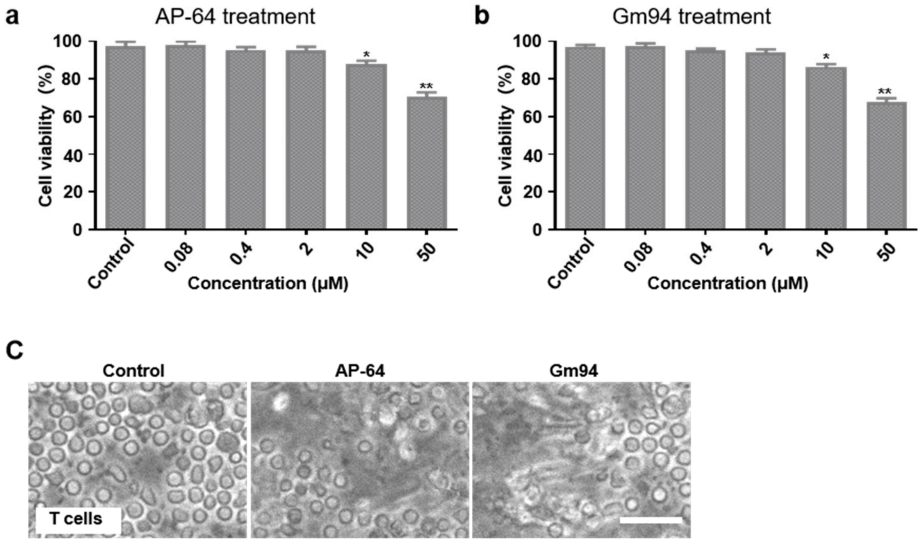

Figure S9. Effect of AP-64 and Gm94 on T cell viability. (a-b) Cell viability assay for T cells. T cells were isolated from the healthy donor peripheral blood mononuclear cells (PBMC) by using beads coated with anti-CD3 and anti-CD28 antibodies (Life Technologies, Grand Island, NY, USA). T cells (5×10^4) were cultured with RPMI 1640 medium containing AP-64 or Gm94 at 37℃ in a humidified atmosphere of 5% CO2. After a 24-h culture, the cell viability was determined use of the CCK-8 assay. All error bars represent SD. * $P < 0.05$, ** $P < 0.01$ (c) Representative images of T cells post treatment with AP-64 or Gm94 at 50 μM for 24 h. Scale bar, 100 μm.

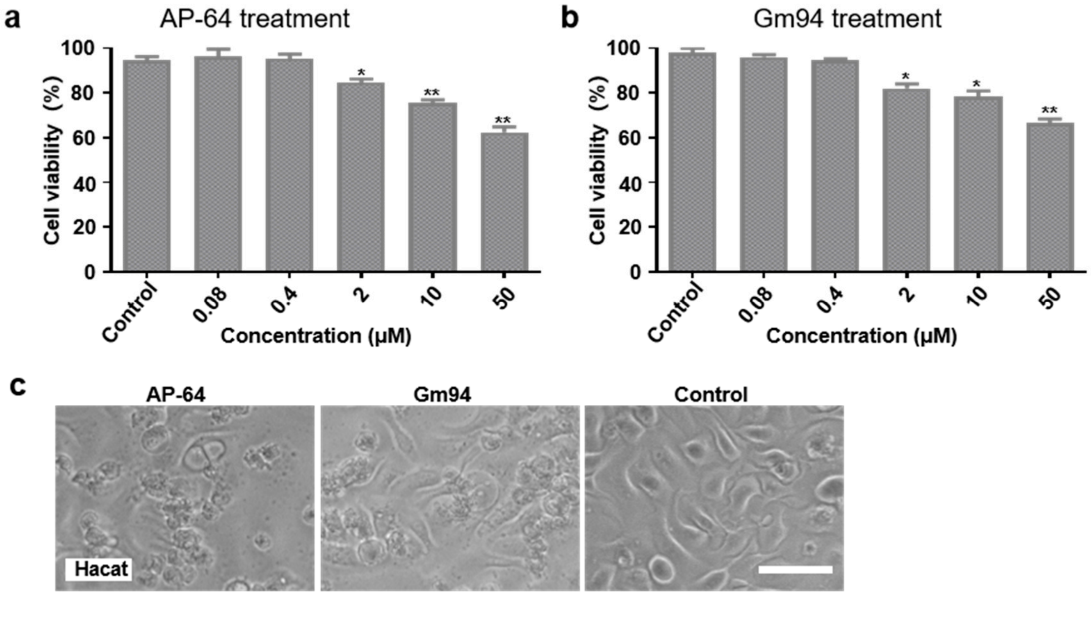

Figure S10. Effect of AP-64 and Gm94 on Hacat cell viability. (a-b) Cell viability of Hacat cells after treatment with the peptides at indicated concentrations for 24 h. All error bars represent SD. * $P < 0.05$, ** $P < 0.01$. (c) Representative images of Hacat cells post treatment with AP-64 or Gm94 at 50 μM for 24 h. Scale bar, 100 μm.

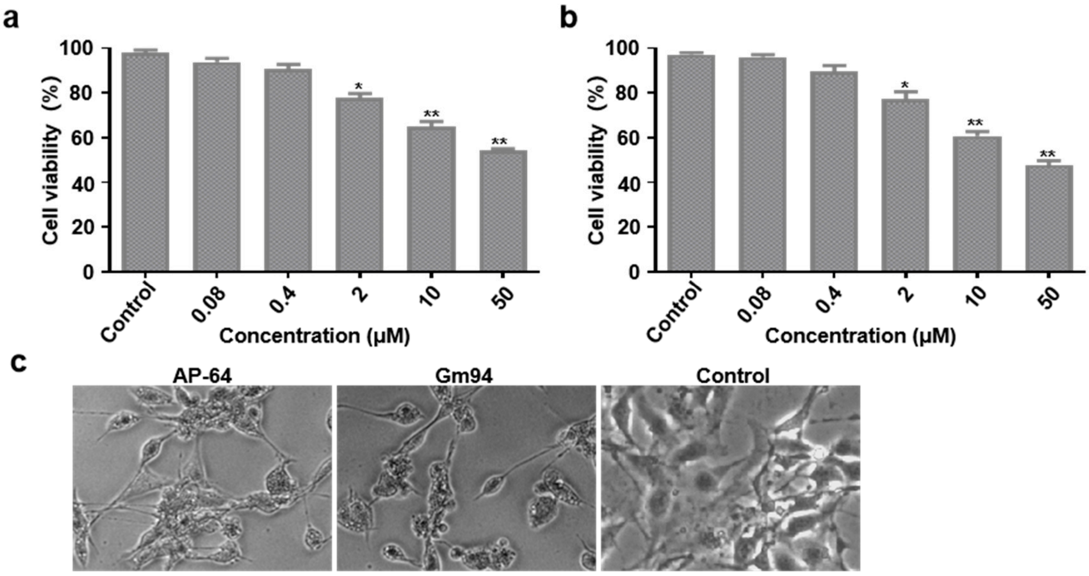

Figure S11. Effect of AP-64 and Gm94 on MEF cells viability. (a-b) Cell viability of MEF cells after treatment with the peptides at indicated concentrations for 24 h. All error bars represent SD. * P < 0.05, ** P < 0.01. (c) Representative images of MEF cells post treatment with AP-64 or Gm94 at 50 μM for 24 h. Scale bar, 100 μm.
